# An epidemiological, strategic and response analysis of the COVID-19 pandemic in South Asia: a population-based observational study

**DOI:** 10.1186/s12889-022-12811-y

**Published:** 2022-03-07

**Authors:** Hafiz Muhammad Salman, Javaria Syed, Atif Riaz, Zouina Sarfraz, Azza Sarfraz, Syed Hashim Abbas Ali Bokhari, Ivan Cherrez Ojeda

**Affiliations:** 1 Service Institute of Medical Sciences, Lahore, Pakistan; 2 Sargodha Medical College, Sargodha, Pakistan; 3 Fatima Memorial Medical and Dental College, Lahore, Pakistan; 4 Fatima Jinnah Medical University, Lahore, Pakistan; 5 Aga Khan University, P.O. Box: 3500, Stadium Road, 74800 Karachi, Pakistan; 6 Universidad Espíritu Santo, Samborondón, Ecuador; ii. Respiralab Research Center, Guayaquil, Ecuador

**Keywords:** South Asia, Urban, Rural, Age, Testing, Vaccine, Case-fatality Ratio, COVID-19

## Abstract

**Introduction:**

South Asia has had a dynamic response to the ongoing COVID-19 pandemic. The overall burden and response have remained comparable across highly-burdened countries within the South Asian Region.

**Methodology:**

Using a population-based observational design, all eight South Asian countries were analyzed using a step-wise approach. Data were obtained from government websites and publicly-available repositories for population dynamics and key variables.

**Results:**

South Asian countries have a younger average age of their population. Inequitable distribution of resources centered in urban metropolitan cities within South Asia is present. Certain densely populated regions in these countries have better testing and healthcare facilities that correlate with lower COVID-19 incidence per million populations. Trends of urban-rural disparities are unclear given the lack of clear reporting of the gaps within these regions. COVID-19 vaccination lag has become apparent in South Asian countries, with the expected time to complete the campaign being unfeasible as the COVID-19 pandemic progresses.

**Conclusion:**

With a redesigning of governance policies on preventing the rise of COVID-19 promptly, the relief on the healthcare system and healthcare workers will allow for adequate time to roll out vaccination campaigns with equitable distribution. Capacity expansion of public health within the Region is required to ensure a robust healthcare response to the ongoing pandemic and future infectious disease outbreaks.

## Introduction

Eight countries constitute South Asia, including Afghanistan, Bangladesh, Bhutan, India, Maldives, Nepal, Pakistan, and Sri Lanka, forming the South Asian Association for Regional Cooperation (SAARC). As of May 10, 2021, this region is responsible for 25.26 million (15.83%) COVID-19 cases and 0.29 million (8.69%) COVID-19 deaths globally [1]. The South Asian Region is home to 23.75% of the global population, and the transmission of COVID-19 in this Region has remained dynamic [2]. Initial projections predicted a higher burden of COVID-19 cases and deaths given the lack of adequate healthcare infrastructure in South Asia [3, 4]. Additionally, highly dense populations within urban settings and overall socioeconomic vulnerabilities across South Asia led to great concern from public health bodies across the globe [5]. Within high-income countries (HICs), many patients admitted to the hospitals and at higher risk of mortality were minority ethnic groups, including South Asians [6]. Various underlying factors that were cited included a higher risk of chronic diseases, especially diabetes and cancer, which increases the relative risk of the hazard ratio for mortality due to COVID-19 [7–9]. The triad of compromised public health infrastructure, under-trained human resources, and contributory environmental factors has been cited as a vector for further viral transmission in the South Asian Region [10, 11].

 South Asian countries share similar socioeconomic backgrounds with fragile health systems. South Asia has previously had gaps in public health preparedness and response to infectious disease outbreaks, notably during the Cholera outbreak. The COVID-19 pandemic has highlighted a lack of a robust infectious control system within this region [12]. The Region's public health is already challenged with a high rate of preventable morbidity and mortality, which requires exploration to aid in the effective management and control of infectious disease outbreaks [11]. In this study, we explored the vulnerabilities in the healthcare system across South Asian countries during the COVID-19 pandemic. We focused on the epidemiology, preparedness, and strategies contributing to the dynamic spread of COVID-19 in this Region. We expanded on specific countries that have had a higher burden of COVID-19 in the Region. Specific factors that we explored included age-distribution patterns, the capacity of COVID-19 testing, vaccination efforts, and prospects across the Region.

## Methodology

**Fig. 1:**
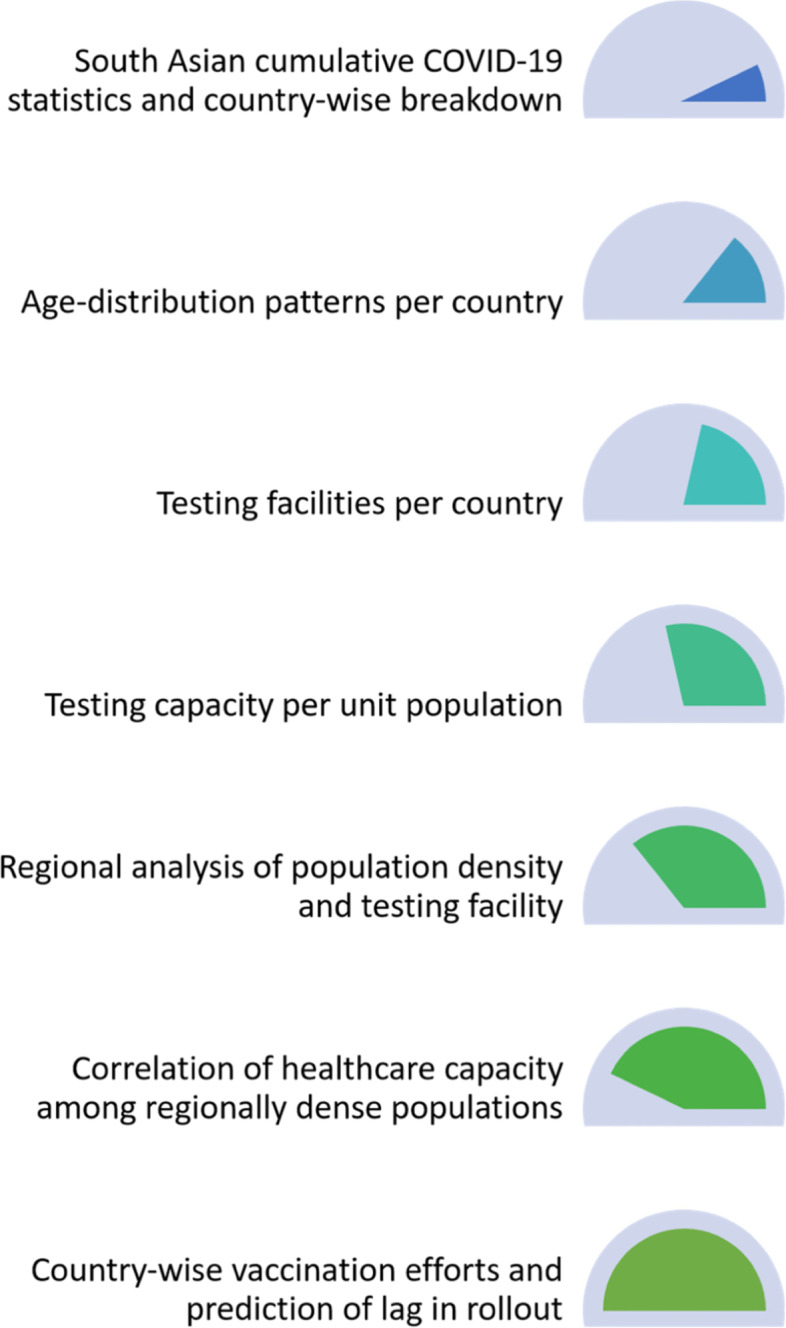
Step-wise breakdown of the employed methodology of epidemiological analysis

Data were monitored between March 1, 2020, to May 19, 2021, to understand the transmission patterns among people within SAARC. A population-based observational survey was conducted by obtaining data from government websites of SAARC (iedcr.gov.bd,covid.gov.pk, corona.mygov.in, covid19.gov.lk, https://heoc.moph.gov.np/updateon-novel-coronavirus-covid-19/moh.gov.bt/novel-coronavirus-2019-ncov/, https://covid19.health.gov.mv/dashboard/af.useembbasy.gov/covid-19-information/) , Statista, Worldometer, the European Center for Disease Control and Prevention (CDC), John Hopkins University of Medicine Coronavirus Resource Center, and the World Health Organization (WHO) dashboard. Population dynamics were obtained from country-specific latest Census Statistics and Statista when applicable. Other sources, including peer-reviewed articles on the MEDLINE and Scopus database, were also reviewed to obtain the latest updates on countries within SAARC. Search terms were used with Boolean operators and included “South Asia”, “pandemic”, “COVID-19”, “Coronavirus”, “response”, “preparedness”, “strategies”, and “healthcare”. Only studies that reported healthcare preparedness and response to the COVID-19 pandemic were included. The current study was exempt from the Institutional Review Board (IRB) due to the secondary analysis of publicly available data. The primary outcome was the correlation of country-wise and regional age distribution, testing facilities and population density, and vaccination rollout efforts within South Asian countries. The secondary outcome was the exploration of gaps in the COVID-19 pandemic to inform healthcare responses within the public health domain. The analysis was conducted using data from government websites with a detailed step-by-step guide attached (Fig. 1).

## Results

### Current Situation in SAARC

Three countries in the Region with the highest popultion have had the highest burden of reported COVID-19 infections, namely India, Pakistan, and Bangladesh. As of May 10, 2021, over 24.63 million cases have been identified among these three countries, which is likely to be underestimated. Screening methods have incorporated airport screening from international travelers with no testing for intra-country travelers. Such screening is beneficial yet does not capture all the patients' incubation periods. Government-imposed social distancing and lockdown measures have been helpful in the Region. However, the testing capacity and facilities have been sub-optimal. The three most impacted countries, India, Pakistan, and Bangladesh, had continued to report high cases in the post-lockdown period after June 2020.

### Age-distribution Patterns

**Fig. 2:**
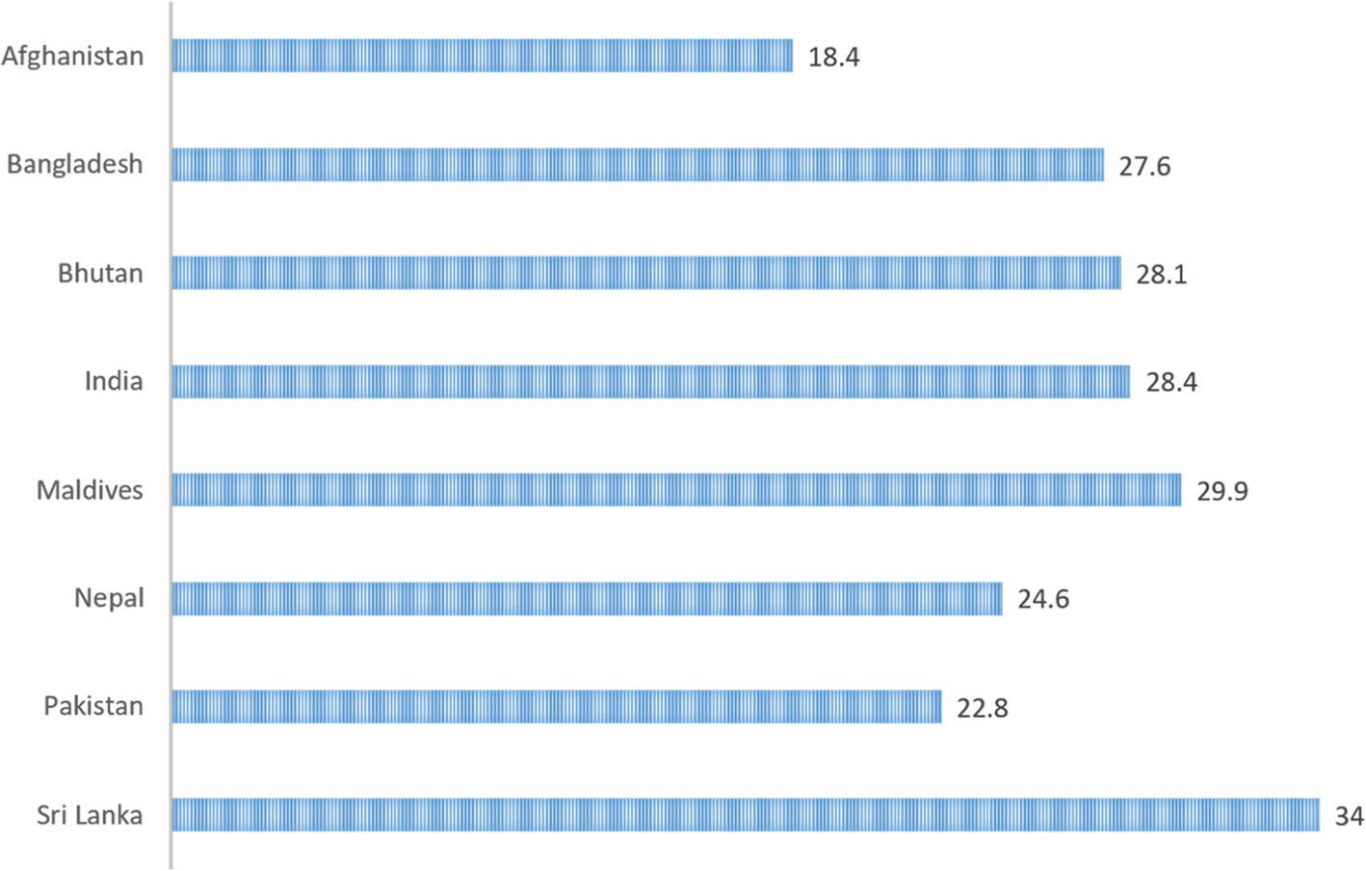
Median Age of SAARC Countries in 2020

The median age of the population in the Region ranges from 22.8 to 34 years in 2020 (Fig. 2). Among SAARC countries, COVID-19 was reported most frequently among the age group below 50 years [13]. Attributed factors include environmental and individual, such as potential ignoring of social distancing protocols to continue working and maintaining employment [10]. Higher incidence in the younger age groups is also likely due to the effective following of social distancing and social welfare programs among older age groups [14]. The case-fatality ratio (CFR) among the SAARC countries was 1.14%, lower than the global CFR of 2.08%. India has the highest burden of deaths within the SAARC countries yet has a CFR of 1.09%, which may be explained by a younger age distribution pattern. Globally, the most vulnerable age group to contract COVID-19 infection is within the age range of 18-64 years. However, the death rate was 45 times higher among 30-39-year-olds and 8,700 times higher among those aged 85 years and older [15]. Given the younger population of the Region, it may be an underlying reason for the relatively lower CFR.

### The Capacity of COVID-19 Testing

**Fig. 3:**
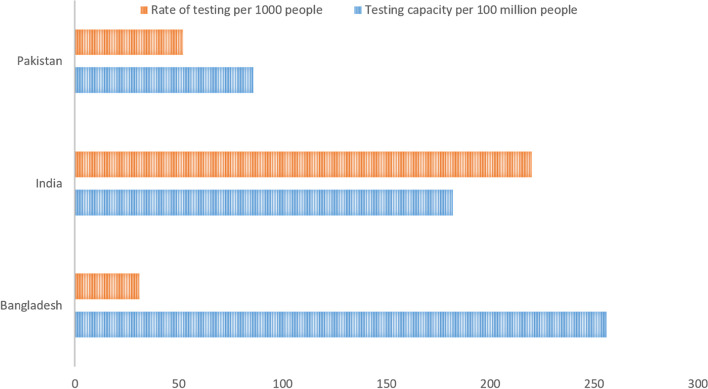
The number of testing centers per 100 million people and the testing rate per 1000 people in the SAARC countries with the highest COVID-19 cases

The testing capacity of SAARC countries has received attention, with Bangladesh being criticized for testing a maximum of 15,000 tests for a population of 165 million [16]. All the SAARC countries with a high positivity ratio expanded their testing centers in the past year. The overall tests conducted in the countries with the heaviest burden of COVID-19 per 100 million people were the highest in India. The number of testing facilities available per 100 million people is demonstrated in Fig. 3. Interestingly, India has performed the highest number of tests per million people compared to their testing capacity, followed by Bangladesh and Pakistan, as demonstrated in Fig. 3. However, the overall burden of COVID-19 remains the highest in India. Across all these countries, there has been a gap of the testing centers in proportion to the population density. There is minimal infrastructure to access rural and remotely-located districts, compounded with social distancing protocols, with greater availability of testing centers primarily in urban and semi-urban settings [17]. There has been a recent spike in the incidence of COVID-19 across the SAARC countries, which has been attributed to the downplaying of the nature of the pandemic, lack of preventive measures taken among citizens, and delayed responses the governments to contain the pandemic. Countries including Bhutan, Maldives, Nepal, and Sri Lanka have performed much better than their regional counterparts due to timely action and effective measures such as social distancing [11].

### Responses to COVID-19

Following the confirmation of the first COVID-19 diagnosed case in each SAARC country, the number of critical days it took for the government to impose a lockdown was variable, with Sri Lanka taking action before its first confirmed case. All the South Asian countries had set a lockdown within one month of the first diagnosed case except India, which implemented the lockdown on March 25, 2020, approximately seven weeks after its first confirmed COVID-19 case. However, South Asia took formidable action quicker than the United States, United Kingdom, and European countries. Despite nationwide lockdowns being implemented between March 15 to 25, 2020, the number of COVID-19 cases have continued to rise. The interventions in South Asia have focused on implementing strict lockdown measures through suppression. There has been a lack of COVID-19 containment among five countries, including Afghanistan, Bangladesh, India, Nepal, and Pakistan. Overall, the CFR in South Asia has been lower than that of the developed countries. However, individual analysis of Pakistan and India places both these countries in the top twenty countries, as per the CFR and deaths per 100,000 populations.

The majority of the heavily-burdened countries in South Asia (India, Pakistan, and Bangladesh) reside in rural areas. The healthcare system in South Asia follows a decentralized system with healthcare provision available through public and private hospitals in three countries (India, Pakistan, and Bangladesh). Limitations of healthcare resources among different states or regions within India, Pakistan, and Bangladesh have been cited as causative for inadequacy to limit community transmission. As of May 10, 2021, India has set up 2,542 testing facilities for its total population of nearly 1.38 billion people. The number of testing centers in Pakistan is 139 for its entire population of 224 million people. Bangladesh received criticism for its inadequate testing facilities and has set up 459 centers for its population of 166 million. There is a significant gap in the capacity of testing centers to serve more populated districts. The number of COVID-19 testing facilities per population density falls short as the most densely populated states have a relatively lower number of testing centers and a higher burden of cases per million populations (Table 1). In the South Asian Region, there has been a shortage of hospital beds per state division. For instance, the number of beds in Islamabad, Pakistan, shows the availability of beds to be 1 for every 38 patients on average per 1 million people. Uttar Pradesh in India and Dhaka in Bangladesh are more competitive due to a stronger healthcare system prior to the COVID-19 pandemic.

**Table 1:** Population density and COVID-19 resources among five districts of India, Pakistan, and Bangladesh. We selected five states with the highest population density. ^a^Not available for each district

Country		Population density per sq. Km.	Number of COVID-19 testing facilities	COVID-19 cases per 1 million people	Number of hospital beds
**India**	Bihar	1,102	65	5,682	30,857
	West Bengal	1,029	128	10,878	113,535
	Kerala	859	159	56,965	99,227
	Uttar Pradesh	828	241	7,525	281,402
	Haryana	573	60	25,248	36,141
**Pakistan**	Islamabad	889	22	98,200	2,571
	Punjab	358	54	4,376	60,387
	Khyber Pakhtunkhwa	238	19	7,084	24,329
	Sindh	216	38	9,679	38,623
	Baluchistan	19	6	3,584	7,797
**Bangladesh**	Dhaka	1,751	454^a^	15,053	131,248^a^
	Mymensingh	1,074		852	
	Rajshahi	1,007		1,754	
	Rangpur	960		1,153	
	Chattogram	831		3,339	

Regardless, the data demonstrated in Table 1 does not account for rural-urban divisions. The testing centers in terms of the highest population and density in India, Pakistan, and Bangladesh highlight a significant gap in the state of Bihar and Kerala in India, and Islamabad and Khyber Pakhtunkhwa divisions in Pakistan. The gap has also been noted in Bangladesh's Dhaka and Mymensingh divisions [16]. There is a centrality of testing centers in urban metropolitan regions with concentrated tertiary healthcare provision. These countries have a higher discrepancy of in-hospital services and testing centers. In India's recent COVID-19 wave, the number of testing centers and hospital bed facilities did not rise in proportion to the number of COVID-19 cases and medical staff and other technical facilities [18]. The healthcare system in these countries has been cited as under-equipped to serve all the population in rural and remote settings, as well as urban settings as the cases rise in the metropolitan areas.

## Vaccination Efforts

**Fig. 4:**
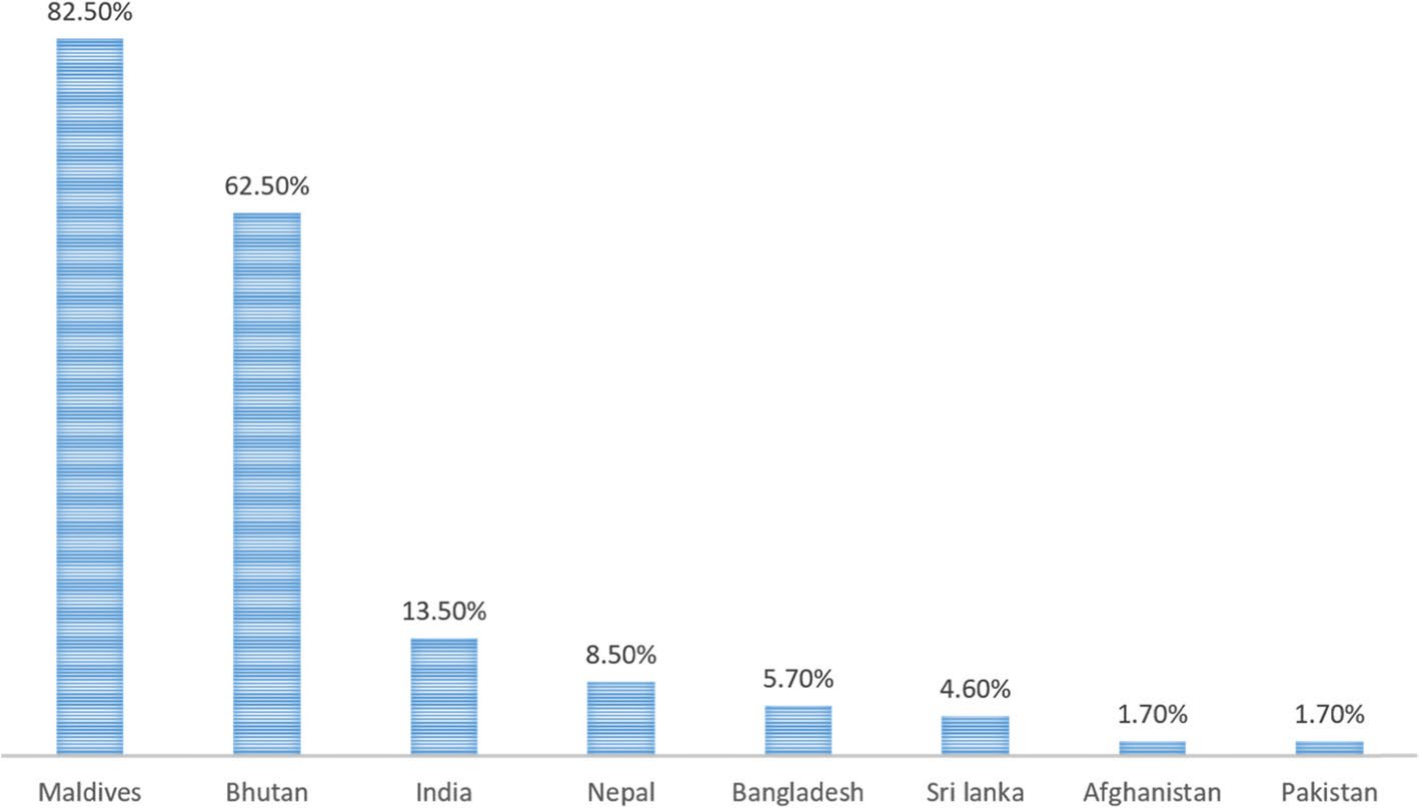
Percentage of the total population vaccinated among SAARC countries until May 19, 2021

Lagging rollout of vaccinations has been noted in the Region as countries with higher burdens, including India, Pakistan, and Bangladesh, relaxed physical restrictions, and detection of new variants of COVID-19. In the early phase of the COVID-19 pandemic in June 2020, the World Health Organization (WHO), Coalition for Epidemic Preparedness Innovations (CEPI), and Gavi, the Vaccine Alliance funded by the Gates Foundation, developed a global initiative to improve access to COVID-19 vaccines [19]. The initiative to distribute two billion vaccine doses throughout 2021 began in February. Further, the pandemic response has been supplemented with aid from the World Bank to aid in vaccination access and rollout in the Region. Of all the South Asian countries, both Maldives and Bhutan have successfully administered the highest vaccinations per percentage of the total population. Maldives and Bhutan vaccinated 5.5% and 8.9% per week, respectively. However, the three countries with the highest burden of COVID-19, namely India, Pakistan, and Bangladesh, had administered an average of 0.8%, 0.1%, and 0.4% per week of the total population. At this rate, India, Pakistan, and Bangladesh require 2.4 years, 19.2 years, and 4.8 years respectively, to vaccinate their entire population. Other South Asian countries, including Afghanistan, Nepal, and Sri Lanka, also lag in the access and rollout of the COVID-19 vaccines, with respective countries requiring 19.2 years, 3.8 years, and 6.4 years. The percentage of the total population having received one COVID-19 dose is summarized in Fig. 4, with the latest estimates until May 19, 2021.

With a substantial amount of vaccination supply provided by COVAX partners, two primary roadblocks need to be addressed during the vaccination campaign by South Asian countries that have lagged. There is an immediate need to increase the vaccine supply and re-strategize the setting for the distribution campaign. For instance, the rural population constitutes 63-65% of the three countries with the highest burden of COVID-19 [20]. However, these settings have a scarcity of public health and primary care clinics [21]. This warrants an equitable distribution through collaboration with primary care facilities and their communities. Alongside, as the vaccination campaign is rolled out, it is pertinent to control the high rate of COVID-19 transmission present in certain South Asian countries. Such containment measures may be possible through transparent prompt reporting of the data by the government, stopping mass religious or political gatherings, tracking hotspots, and necessitating masks and social distancing.

## Discussion

There has been a notable difference in the testing strategies, laboratory strategies, and healthcare provision among and within South Asian countries [11]. The insufficient facilities and the ensuing community transmission during the early stage of the pandemic have not been met with adequate governance in the region due to premature and discontinuation of social distancing policies [22]. So far, South Asia has been observed to have lower CFRs which may be underestimated. The CFR of Pakistan is the highest in the region, yet Pakistan had the most insufficient testing capacity in the Region, as noted in our analysis. However, in the initial phase of the COVID-19 pandemic, Pakistan's CFR was significantly lower, which is suggestive of the inadequate surveillance system. With a growing number of cases, the CFR has increased in Pakistan, and it presents two possible dilemmas: 1) the number of individuals being tested is more severely ill, resulting in an overestimation of CFR, and 2) the number of mild-to-moderate COVID-19 cases are under-represented due to under-testing of patients. A bias noted in the trends of CFR in the Region may be present, yet there is notable ongoing improvement in the testing capacity. 

Following trajectories of lockdown and post-lockdown trends in the SAARC countries, smaller countries were able to control their COVID-19 transmission. However, the three most affected countries (India, Pakistan, and Bangladesh) eased lockdowns without curbing the transmission of COVID-19 cases. Before recommending nationwide lockdowns during the ongoing wave of the COVID-19 pandemic, certain highly dense hotspots need to be considered, such as Mumbai, Karachi, and Dhaka, the three most populated urban cities in India, Pakistan, and Bangladesh [11]. Selective hotspots require localized lockdowns for 2-3 weeks with active surveillance and strict quarantine measures to prevent a severe outbreak in these countries. A public health approach necessitates curbing the peak (the exponential growth in COVID-19 infection requiring medical supplies) a few weeks before it occurs. Gaps in response preparedness in the Region include shortages in hospital beds, ventilators, quarantine facilities, and a lack of standardized treatment protocols due to different variants, which present delays in health provision [12]. SAARC countries have received testing kits, ventilators, vaccinations, and aid from international regulatory bodies. However, the overall health expenditure in the three most impacted and populated countries (India, Pakistan, and Bangladesh) is less than 1% of GDP [23]. The total availability of hospital beds is lower than 10 per 1000 populations in all the SAARC countries except Bhutan [24]. Further, the World Bank has predicted that SAARC countries will face their worst economic crisis due to the ongoing COVID-19 pandemic.

Within the socioeconomic context of the South Asian Region, the health systems have already witnessed a scarcity of healthcare resources. The COVID-19 pandemic further aggravated the gaps in healthcare resources [17]. The pressure to ease lockdowns was also felt due to the economic losses faced by these countries. In the South Asian context, the lack of clarity within the urban-rural disparity in testing, vaccination access and acceptance, and treatment presents a challenge. High-burden countries (India, Pakistan, Bangladesh, Sri Lanka, and Afghanistan) in the Region have reported shortages in medical supplies such as oxygen, hospital beds, and personal protective equipment (PPE) [25]. The detection of emerging strains was compounded by a lack of mitigation strategies and the country's catastrophic second wave of COVID-19 [18]. With the Region, it is also pertinent to eliminate false reports [26]. Misinformation concerning COVID-19 infection and vaccination needs to be addressed through education, catering to eliminating vaccine hesitancy and promoting the application of safety protocols (masks, distancing) [27].

A limitation of the available data from the government sites is the potential underestimation of the actual scenario. For instance, data quality and confounders were not elucidated at public repositories. Also, as the current study focuses on a population-level analysis, we were unable to explore individual-level factors. Regardless, this study focuses on estimating country-level representative data that may be generalizable for public health bodies at a national level.

### Future Scope of the Work

The SARS-CoV-2 virus has been identified with a large number of variants, and there is uncertainty regarding its transmissibility, immune system evasion, and severity. The new variant B.1.1.529, named Omicron, was detected on November 26, 2021, and it is being considered a variant of concern as cases are spiking globally [28]. As of December 31, 2021, 6 countries in the South Asian Region, including India, Pakistan, and Bangladesh, which have had the highest burdens in previous waves, have reported confirmed Omicron cases. Within the Region, countries have previously experienced a few waves of the COVID-19 pandemic. All three previously high-burdened countries, including India, Pakistan, and Bangladesh, have 40%, 31%, and 32% of their population primarily vaccinated. Booster doses of mRNA vaccines are also being administered in the Region, with preliminary results suggesting a 20-40-fold reduction in neutralizing antibody activity for Omicron [29]. The severe shortfall in vaccine supply across the region is likely to predispose underprivileged communities within these countries to the Omicron outbreak [30]. These gaps are especially pronounced in rural settings as the majority of the information-sharing mechanisms are available online [31, 32]. Further, appropriate public health measures to mitigate risk are needed including vaccination; however, this may not be sufficient [33]. There is rising concern that emerging strains may reduce the immunity of previously vaccinated individuals.

The recommended course with the rise of Omicron is to accelerate vaccination of high-priority groups and strengthen health systems. The COVID-19 pandemic has highlighted the vulnerabilities of the healthcare system in the Region. The lack of robust public health infrastructure and personnel was apparent, and there is an urgent need to address the health systems disparities in the Region [34]. Countries with higher COVID-19 cases in the region have expanded their expenditure on capacity building in healthcare [35]. However, countries with better healthcare structures, less poverty, and a younger population in the Region have performed similarly to their counterparts [35]. India has played a critical role in setting the emergency fund for the public health expenditure of SAARC countries [36]. Even as SAARC addresses challenges in its public health system, there is an urgent need for a stronger healthcare investment to promote health security. SAARC needs to act in unison through pooled resources to improve vaccine coverage, access to medicines, PPEs, and other equipment and services [37]. The public health infrastructure may focus on improving efficiencies by introducing online disease surveillance systems, telemedicine, and point-of-care tests, which are already in use in developed countries. Similarly, effective mitigation strategies include strict quarantine measures, equipped diagnostic facilities, and public education. As the region shares similar challenges in public health, it is pertinent to consider a collated, cost-effective plan to combat the ongoing COVID-19 pandemic and future infectious disease outbreaks.

## Conclusion

- The challenges to the COVID-19 outbreak in the Region are similar due to weak healthcare systems and comparable socioeconomic backgrounds.
- The urban-rural disparities have not been documented in national datasets despite a significant ratio of the population belonging to rural settings, with services not reaching these remote and inaccessible areas.
- Trends of COVID-19 burden in more populated states/territories are associated with an overwhelmed healthcare system, service provision gaps, and limited medical equipment and personnel primarily due to economic and capacity challenges.
- Vaccination rollouts in the Region have been met with difficulty due to vaccine shortages, inequitable distribution globally, vaccine hesitancy, and the low health capacities of the countries.
- Several actions taken in the previous waves of the COVID-19 pandemic have promoted the capacity building of public health in the Region; yet, the public health capacity requires further expansion within and across the Region's countries.
- Persistent gaps in healthcare provision must be addressed to expand the provision of health services with the ongoing Omicron variant and upcoming infectious disease outbreaks.

## Data Availability

All data was sourced from public repositories.
